# Regenerative Effects of CDP-Choline: A Dose-Dependent Study in the Toxic Cuprizone Model of De- and Remyelination

**DOI:** 10.3390/ph14111156

**Published:** 2021-11-12

**Authors:** Viktoria Gudi, Nora Schäfer, Stefan Gingele, Martin Stangel, Thomas Skripuletz

**Affiliations:** Department of Neurology, Hannover Medical School, 30625 Hannover, Germany; gudi.viktoria@mh-hannover.de (V.G.); sylnora@t-online.de (N.S.); gingele.stefan@mh-hannover.de (S.G.); Stangel.Martin@mh-hannover.de (M.S.)

**Keywords:** multiple sclerosis, CDP-choline, cuprizone, microglia, astrocytes, oligodendrocytes

## Abstract

Inflammatory attacks and demyelination in the central nervous system (CNS) are the key factors responsible for the damage of neurons in multiple sclerosis (MS). Remyelination is the natural regenerating process after demyelination that also provides neuroprotection but is often incomplete or fails in MS. Currently available therapeutics are affecting the immune system, but there is no substance that might enhance remyelination. Cytidine-S-diphosphate choline (CDP-choline), a precursor of the biomembrane component phospholipid phosphatidylcholine was shown to improve remyelination in two animal models of demyelination. However, the doses used in previous animal studies were high (500 mg/kg), and it is not clear if lower doses, which could be applied in human trials, might exert the same beneficial effect on remyelination. The aim of this study was to confirm previous results and to determine the potential regenerative effects of lower doses of CDP-choline (100 and 50 mg/kg). The effects of CDP-choline were investigated in the toxic cuprizone-induced mouse model of de- and remyelination. We found that even low doses of CDP-choline effectively enhanced early remyelination. The beneficial effects on myelin regeneration were accompanied by higher numbers of oligodendrocytes. In conclusion, CDP-choline could become a promising regenerative substance for patients with multiple sclerosis and should be tested in a clinical trial.

## 1. Introduction

Multiple Sclerosis (MS) is a chronic demyelinating autoimmune disease of the central nervous system (CNS) leading to neuronal damage resulting in functional disability. Remyelination is the natural regenerating process of demyelinated nerves and frequently occurs after demyelinating events in all manifestations of the disease [1,2,3,4,5]. However, this repair process is not an invariant response to demyelination and is often incomplete or even fails in MS patients [6,7]. Moreover, remyelination seems to be very heterogeneous between individual patients and lesions [8]. Evidence from experimental models and insights from MS tissue revealed that the extent of remyelination shows an age-related decline and depends on disease duration [9,10,11,12,13]. Numerous animal studies provided several lines of evidence that remyelination could protect axons from degradation and might lead to functional recovery and restoration of axonal conduction velocity [14,15,16]. Imaging studies in MS patients and brain tissue analyses showed functional activity and axonal preservation in remyelinated areas [8,17,18].

Currently available disease-modulating therapies primarily address the immune system and target mostly a single molecule or pathway on/in different subtypes of immune cells and block the entry of these cells in the CNS or deplete different immune cells such as B or T cells [19,20,21,22,23]. Findings from MS tissue and a number of experimental studies highlighted that immune cells not only drive demyelination and inflammation but also produce molecules that negatively affect remyelination [24,25,26,27]. Transfer of myelin-reactive Th-17 cells impaired endogenous remyelination in cuprizone animal model for MS [28]. Nowadays, the growing body of evidence from different experimental models suggests that immune cells could possess beneficial effects on remyelination by promoting proliferation and differentiation of oligodendrocytes and phagocytosis of disrupted myelin [29,30,31,32]. However, inflammation seems to be not the sole power of neurodegeneration and accumulating disability since MS progression does not correlate with relapses frequency, and disease-modulating drugs are insufficient in preventing neurological disability during the progressive stage of MS [20,33]. To date, potent therapeutic agents to enhance remyelination or neuroprotection are not available in humans.

Cytidine-S-diphosphate choline (CDP-choline) is a naturally occurring endogenous nucleoside and a precursor of phospholipid phosphatidylcholine (PC), which is the major structural component of biomembranes and the source of bioactive lipids in eukaryotic cells. The CDP-choline pathway of PC biosynthesis was described in the 1950s by Eugene P. Kennedy and co-workers and is, therefore, known as the Kennedy pathway [34,35]. Exogenously supplied CDP-choline is sequentially hydrolyzed and dephosphorylated to cytidine (or uridine in humans) and choline. Choline is absorbed rapidly, resynthesized in the brain, and efficiently utilized for membrane lipid synthesis or contributes to the formation of nucleic acids, proteins, and acetylcholine [36]. In various animal models of CNS disorders, CDP-choline enhanced membrane repair and neuronal functions [37,38]. Unfortunately, the promising neuroprotective effects of CDP-choline in animal models of stroke and brain injury could not be reproduced in humans [39,40]. However, since in stroke and brain injury there is a completely different mechanism of lesion induction, the results cannot be simply transferred to demyelinating disorders such as MS. MS lesions are accompanied by demyelination, while most axons are not completely damaged during the onset of disease, although defined axonal loss may occur already in the early stages of the disease [41,42,43]. Inflammation-related cytokines are known to induce disturbances in mitochondrial functions and in axonal transport leading to an accumulation of synaptic vesicles, visualized by amyloid precursor protein (APP) or synaptophysin immunohistochemical stainings [44,45,46,47,48]. In a previous study using the murine cuprizone model, we observed that synaptophysin positive spheroids occurred mostly at the peak of demyelination and microglia accumulation [48]. However, at the onset of remyelination, small and middle-sized spheroids disappeared, indicating a possible recovery of axonal functions, and only big ovoids in probably dissected axons remained for a long time despite ongoing remyelination [48]. The presence of intrinsic regeneration mechanisms in axons and the possible reversibility of axon damage were also observed by other research groups [17,49,50]. Neurodegeneration seems to be rather a slow process triggered by different pathological events, such as oxidative stress, mitochondrial dysfunctions, disarrangement of different ion channels due to prolonged demyelination, or generally increased vulnerability to repeated inflammatory events [51,52]. Therefore, besides a reduction of inflammation, remyelination promoting therapies are needed to reduce neurodegeneration.

Another possible explanation for the different results of CDP-choline action in preclinical and clinical studies could be simple physiological differences between humans and animals. Moreover, extrapolated from MS research, no single experimental model covers the entire spectrum of the clinical, pathological, or immunological features of the disease [53]. However, appropriate experimental models still provide a solid platform for the investigation of distinct aspects of the disease. In our study, we applied a well-established and reliable animal model of toxic demyelination. This model is known to be particularly useful for studying remyelination [54,55].

Previously we have shown that the food supplement CDP-choline (500 mg/kg body weight per day) enhanced remyelination in two different animal models of CNS demyelination, demonstrating a new mechanism via increased proliferation of oligodendrocyte progenitor cells (OPC) during the remyelinating process [56]. In addition, the contrasting results in animals and humans may be due to the large differences in the dosage of drugs used in the two approaches. In animal experiments, usually 500 mg/kg CDP-choline was applied, which would be equivalent to 40 g CDP-choline per day. However, in clinical trials, considerably lower doses of 0.5–2 g per day were used [57,58]. Thus, the aim of our current experiments was to investigate whether lower doses of CDP-choline that are equivalent to doses used in human clinical trials might be sufficient to enhance remyelination in a murine model of demyelination.

## 2. Results

### 2.1. Different Doses of CDP-Choline Improved Remyelination after Cuprizone Induced Demyelination

**Figure 1:**
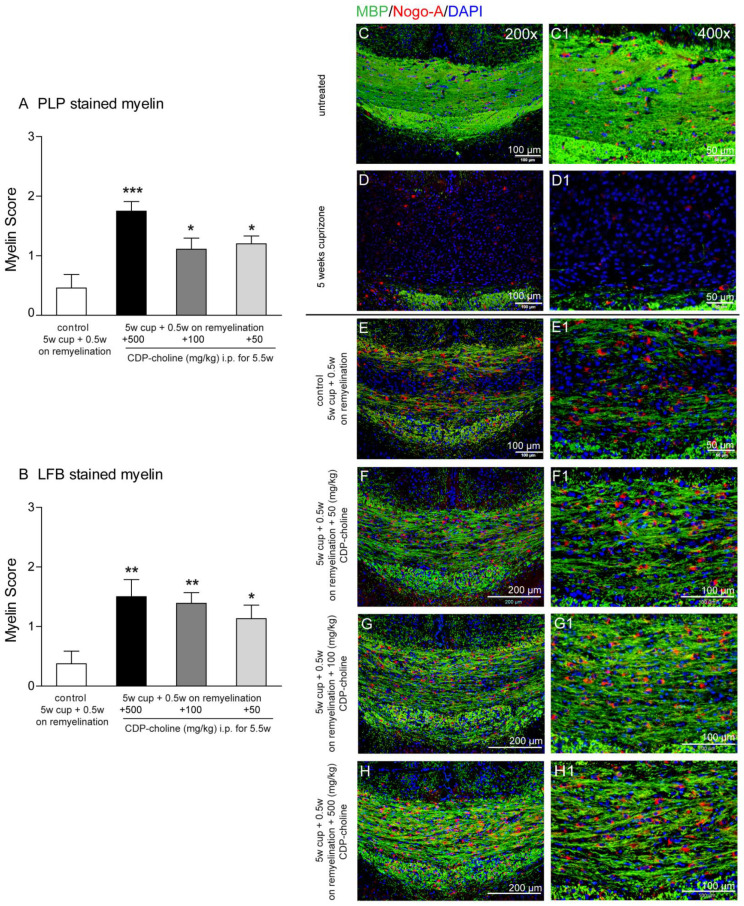
Graphs evaluate initial remyelination (week 5.5—half a week on remyelination) in the corpus callosum after 5 weeks of 0.2% (*w/w*) cuprizone-containing diet (complete demyelination). Animals received different CDP-choline doses (50 mg per kg; 100 mg per kg; 500 mg per kg, intraperitoneally) or sham (PBS) once a day for the duration of the whole experiment. In (**A**), the re-expression of myelin protein PLP is shown. Graph (**B**) demonstrates the re-appearance of lipid phase of new myelin stained with LFB. Sections from 6 animals per group were scored by three blinded independent observers. Score 3 corresponds to normal myelin appearance, whereas score 0 means complete demyelination. Regardless of the dosage applied animals treated with CDP-choline showed a significantly increased re-expression of myelin protein PLP as well as LFB as compared to the control group (5 weeks cuprizone + 0.5 weeks on remyelination, PBS). Representative images (**C**–**H1**) illustrate re-expression of myelin protein MBP visualized in green and Nogo-A positive oligodendrocytes (in red) presented in the remyelinating (week 5.5) corpus callosum of mice exposed to different dosages of CDP-choline (**G**–**I**) or PBS. (**C**) depictures the corpus callosum of untreated animals, whereas the completely demyelinated corpus callosum is showed in (**D**). (**C1**–**H1**) images provide a higher magnification of area, showed in (**C**–**H**). Significant effects between mice treated with different dosages of CDP-choline and PBS are indicated by asterisks (* *p* < 0.05, ** *p* < 0.01, *** *p* < 0.001).

Feeding cuprizone for 5 weeks induced a nearly complete loss of myelin proteins in the corpus callosum, as shown by the staining for the myelin marker myelin basic protein, (MBP) (Figure 1D,D1). After cessation of cuprizone from the diet, remyelination occurred, and first myelin sheaths were visible in the corpus callosum at the time point of 5.5 weeks, which represents early remyelination (Figure 1E,E1). Animals treated with CDP-choline in different doses (500, 100, and 50 mg/kg body weight) showed significantly higher re-expression of myelin protein myelin proteolipid protein (PLP) (Figure 1A, *p* = 0.003) as compared to the control group (5 weeks cuprizone + 0.5 weeks on remyelination, no CDP-choline). Similar results were found by analyzing the histochemical luxol-fast blue (LFB) staining of myelination (Figure 1B) and MBP stained myelin (Figure 1E–H1). Mice treated with the highest dose of CDP-choline (500 mg/kg body weight) tended to show higher remyelination as compared to mice that received CDP-choline at lower doses (100 and 50 mg/kg body weight). Statistical analysis revealed no significant difference between the CDP-choline groups, neither for the immunohistochemical myelin protein stainings nor for the histochemical LFB staining.

### 2.2. CDP-Choline Enhanced the Numbers of Oligodendrocytes during Early Remyelination

**Figure 2:**
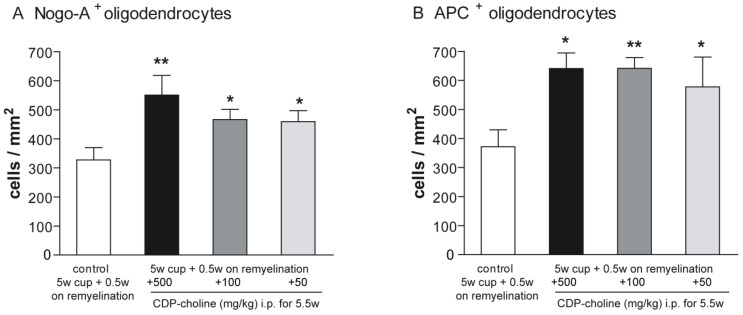
Differentiation and maturation of oligodendrocytes were visualized with Nogo-A and APC stainings. Graphs demonstrate the number of Nogo-A positive cells (**A**) and APC positive cells (**B**) in per mm^2^ (n = 6). Mice that received CDP-Choline at doses of 500 mg/kg, 100 mg/kg, and 50 mg/kg body weight, respectively, showed significantly increased numbers of adult oligodendrocytes as compared to the control group (no CDP-choline). Significant effects between mice treated with different dosages of CDP-choline and PBS are indicated by asterisks (* *p* < 0.05, ** *p* < 0.01).

Mature oligodendrocytes were visualized using the established immunohistochemical markers Nogo-A and adenomatus polyposis coli (APC). Evaluation of the Nogo-A and APC staining at early remyelination revealed that mice treated with CDP-choline at doses of 500 mg/kg, 100 mg/kg, and 50 mg/kg body weight, respectively, showed significantly increased numbers of mature oligodendrocytes as compared to controls (for both marker *p* = 0.02, Figure 1C–H and Figure 2). There were no significant differences in the numbers of mature oligodendrocytes between doses in both stainings.

### 2.3. Elevated Remyelination Was Accompanied by Decreased Microglia Numbers in CDP-Choline Treated Animals

**Figure 3:**
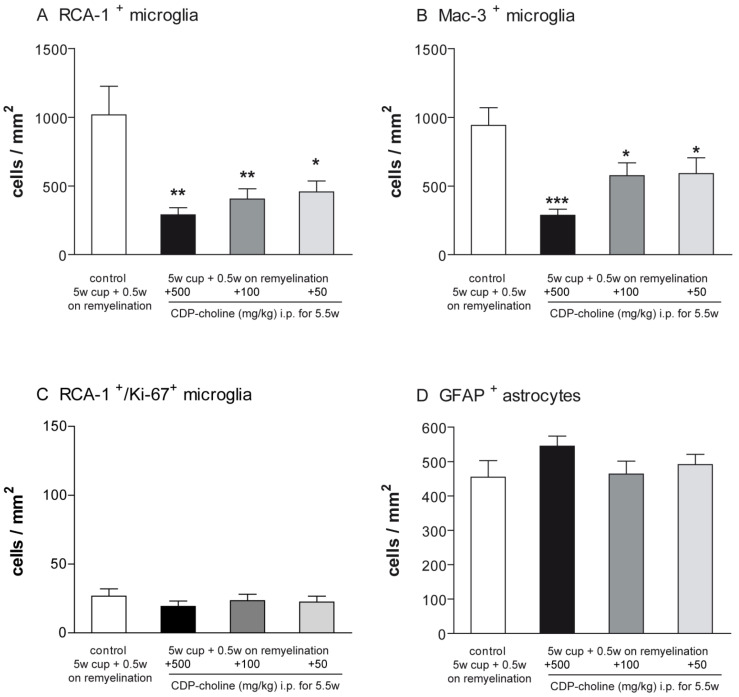
Graphs represent the qualitative analysis (cells/mm^2^) of activated microglia ((**A**)—RCA-1 positive microglia; (**B**)—Mac-3 positive microglia, (**C**)—proliferating RCA-1/Ki-67 positive microglia), and GFAP positive astrocytes (**D**) during early remyelination (week 5.5) in the corpus callosum of animals treated with different dosages of CDP-choline or sham (PBS). The astrogliosis was not affected by CDP-choline treatment. However, the number of activated microglia in all animals treated with CDP-choline, regardless of the dose used, was significantly lower than in the control group. Significant effects between mice treated with different dosages of CDP-choline and PBS are indicated by asterisks (* *p* < 0.05, ** *p* < 0.01, *** *p* < 0.001, n = 6).

Activated microglia were studied using the Mac-3 and ricinus communis agglutinin 1 (RCA-1) markers. The evaluation of microglial numbers at week 5.5 revealed significantly lower numbers of activated microglia in all three animal groups treated with CDP-choline compared to cuprizone controls (for RCA-1 *p* < 0.01, for Mac-3 *p* = 0.008, Figure 3). Mice that received CDP-choline at a dose of 500 mg/kg and 100 mg/kg body weight tended to have a lower number of activated microglia as compared to animals treated with CDP-choline at a dose of 50 mg/kg body weight. However, this difference was not significant, neither for the Mac-3 nor for the RCA-1 staining (Figure 3A,B).

Proliferating microglia were assessed using immunofluorescence double staining with the microglia marker RCA-1 and the proliferation marker Ki-67. No significant difference could be found between the animals treated with CDP-choline at different doses and the control group at the time point investigated (Figure 3C).

Astrogliosis was investigated using antibodies against the glial fibrillary acidic protein (GFAP). The number of GFAP positive astrocytes did not change in accordance with CDP-choline treatment, regardless of the dosage applied (Figure 3D).

## 3. Discussion

Here, we investigated whether the regenerative effect of CDP-choline previously shown in a toxic model of MS, might be dose-dependent. In animal experiments, usually higher doses of CDP-choline were used, preferentially 500 mg/kg body weight. The CDP-choline dose used in clinical trials varies between 500 and 2000 mg per day (preferentially 1000 mg), which corresponds to 6–25 mg/kg for 80 kg person [57,58]. This huge difference in the dose of CDP-choline applied to animals in preclinical studies as compared to human dosing used in clinical trials may be a reason for the irreproducibility of some preclinical results in a clinical setting. However, the dosing used in animal experiments should not be extrapolated to the human dose by a simple conversion according to body weight. For the more appropriate conversion, Reagan-Shaw and colleagues suggested using the body surface area (BSA) normalization method. BSA correlates well across several mammalian species with several parameters of biology, including oxygen utilization, caloric expenditure, basal metabolism, blood volume, circulating plasma proteins, and renal function [59,60]. Furthermore, the US Food and Drug Administration (FDA) recommended in “Guidance for Industry Estimating the Maximum Safe Starting Dose in Initial Clinical Trials for Therapeutics in Adult Healthy Volunteers” using the body surface area conversion factor (BSA-CF) for deriving a human equivalent dose (HED) [61]. Thus, using the formula: animal mg/kg body weight dose x animal k_m_ (3 for mice) ÷ human k_m_ (37 for 60 kg human person) = human mg/kg body weight dose [59,61], where k_m_ is a species-specific conversion factor equal to the body weight in kg divided by the surface area in m^2^, we will receive a human dose of approximately 41 mg/kg body weight corresponding to the 500 mg/kg body weight CDP-choline dose for mice. If we calculate the single daily dose of CDP-choline, we obtain a value of about 3300 mg for an 80 kg person, which still appears quite high. On the other hand, if we convert 100 mg/kg and 50 mg/kg dose in our animal study to the corresponding human dose using the BSA method, we obtain CDP-choline doses that are similar to those given in most clinical trials (50 mg/kg and 100 mg/kg dose in mice corresponds to 324 mg or 650 mg per day, respectively, for an 80 kg person) [57].

Since the high doses (500 mg/kg) cannot be applied in human trials, further animal experiments were needed. Because of our calculations, we decided to investigate the effects of 100 and 50 mg CDP-choline per kg body weight i.p. on myelin regeneration in mice. Intraperitoneal injection of CDP-choline is a well-established and frequently used treatment protocol in various animal species (rabbit, rat, mouse) [37]. We have found that even lower doses of CDP-choline (100 and 50 mg/kg) were able to promote remyelination after toxin-induced demyelination. On the one side, we could replicate our previous results showing beneficial regenerative effects of CDP-choline by using the described dose of 500 mg/kg [56]. On the other side, we could show that even a lower dose of 50 mg/kg was still able to promote faster myelin re-expression. This result is of particular interest because the low dose of CDP-choline more closely matches doses commonly used as oral administration in human studies. It raises the possibility to apply the low dose in a clinical trial in MS patients aiming to prove possible beneficial effects on remyelination and thus neuroprotection. Concomitant to better remyelination, higher numbers of mature oligodendrocytes were identified in the corpus callosum as a consequence of treatment with CDP-choline. The results confirm our previous observations as we have shown that CDP-choline enhanced remyelination in two different animal models of CNS demyelination, demonstrating a new mechanism via increased proliferation of OPC resulting in higher numbers of mature oligodendrocytes leading to faster remyelinating process [56]. In the current study, we observed a significantly elevated re-population of the demyelinated corpus callosum with newly differentiated oligodendrocytes even upon the treatment with the lowest doses of CDP-choline. We also investigated the proliferation of OPC at the onset of remyelination (data not shown). However, at this time point, we could not detect any significant difference between CDP-choline treated and control animals. We suggest that we did not investigate the correct time window, and the peak of OPC proliferation has already passed. Microglia and astrocytes are strongly involved in remyelination and could display detrimental but also beneficial effects on this process [62]. CDP-choline was shown to affect astrocytic cell cycle/differentiation and increase transglutaminase activity in vitro [63,64]. In addition, CDP-choline caused an elevated glutamate uptake and an increased membrane expression of EAAT2 glutamate transporter in cultured rat astrocytes, thus providing neuroprotective effects [65]. In the study using the traumatic brain injury (TBI) zebrafish model, CDP-choline was shown to possess neuroprotective and regenerative functions and increased the production of anti-inflammatory and phagocytoses-promoting cytokines in microglia [66]. Thus, an important question addressed in our work was the possible effect of CDP-choline treatment on astrocytes and microglia. However, we found that CDP-choline did not change cuprizone-induced astrogliosis, at least the numbers of activated GFAP positive astrocytes did not differ. In our previous study, we could not detect any direct effects of CDP-choline on cultured primary microglia [56]. In contrast, in the cuprizone model, CDP-choline reduced the numbers of activated microglia during remyelination. It can be assumed that CDP-choline does not exert its effects directly on activated microglia, but the accelerating remyelination directly leads to an earlier decrease in activated microglia. The disappearance of activated microglia after cuprizone-induced demyelination is probably explained by the self-induced cell death of microglia by released signals from oligodendrocytes or astrocytes and serves as a form of autoregulation to limit the microglial reaction and allow regeneration after damage stimulus [67,68,69].

Numerous clinical studies confirmed a beneficial safety profile of CDP-choline in preclinical animal studies and clinical trials. The median lethal dose (LD50) of a single intravenous dose of CDP-choline is 4600 mg/kg in mice and 4150 mg/kg body weight in rats [70]. In humans participating in clinical trials, only a few mild, transient adverse effects related to gastrointestinal discomfort, malaise, headache, and irritability have been reported, while no data are available on liver or kidney failure [71,72,73,74]. The bioavailability of CDP-choline administrated upon oral and intravenous routes is similar [75]. After injection, CDP-choline is rapidly metabolized to its cholinergic and pyrimidinergic catabolites, being probably even safer and a more “procognitive” form of choline supplement [76,77,78,79,80,81]. Nevertheless, there are still a lot of open questions regarding the place and particular mechanisms of CDP-choline catabolism and its precise mode of action as well its involvement in some kinases activation and trimethylamine N-oxide (TMAO) formation [58,76]. The best way to prove the efficacy of CDP-choline should be to investigate the lowest dose of the drug that still possesses its regenerative efficacy.

## 4. Materials and Methods

### 4.1. Animal Experiments

Experimental toxic demyelination was induced by feeding male 8–10-week-old mice a diet containing 0.2% cuprizone (bis-cyclohexanone oxaldihydrazone, Sigma-Aldrich Inc., USA) mixed into a ground standard rodent chow [62]. The cuprizone diet was maintained for 5 weeks. Then, animals were fed with a normal rodent chow for an additional half a week. Animals received different CDP-choline doses (50 mg/kg body weight; 100 mg/kg body weight; 500 mg/kg body weight, intraperitoneally) or sham (PBS) once a day during the entire experiment, n = 6. Additional control animals were fed with normal chow and received CDP-choline or sham injections as described [56].

All research procedures were approved by the Review Board for the Care of Animal Subjects of the district government and performed according to international guidelines on the use of laboratory animals. Male *C57BL/6* mice were purchased from Charles River Laboratories (Sulzfeld, Germany).

### 4.2. Histology and Immunohistochemistry

Tissue processing was performed as previously described [82]. At week 5.5, a time point of early remyelination, deeply anaesthetized mice were perfused transcardially with 4% paraformaldehyde (PFA, Merck). Brains were removed, postfixed in 4% PFA, and paraffin-embedded. There were 7 μm serial coronal sections cut on a bright rotary microtome (RM2245, Leica) from −0.82 mm bregma to −1.70 mm bregma. A group size of 4 to 6 animals was evaluated immunohistochemically. Histology for Luxol-fast blue periodic acid-Schiff base (LFB-PAS) and immunohistochemistry were performed as previously described [56]. For immunohistochemistry, paraffin-embedded sections were dewaxed and heat-unmasked in 10 mM citrate buffer (pH 6.0). The following primary antibodies were used: for myelin proteolipid protein (PLP) (mouse monoclonal IgG2a, 1:500, Serotec) and myelin basic protein (MBP) (mouse monoclonal IgG2b, 1:500, Covance), for activated microglia Mac-3 (rat IgG, 1:500, BD Pharmingen) and the lectin ricinus communis agglutinin 1 (RCA-1) (1:1000, biotinylated, Vector Laboratories), for astrocytes glial fibrillary acidic protein (GFAP) (mouse IgG, 1:200, Millipore), for oligodendrocytes Nogo-A (rabbit IgG, 1:750, Millipore) and anti-adenomatus polyposis coli (APC; mouse IgG; 1:200).

### 4.3. Determination of Remyelination and Quantification of Glial Reactions

The extent of remyelination was studied as described previously. Sections stained for myelin proteins (PLP, MBP) or Luxol-fast blue solution were scored in a blinded manner by 3 observers in the midline of the corpus callosum on a scale from 0 (complete demyelination) to 3 (normal myelin) using a light microscope (Olympus BX61). Glial reactions were investigated on the basis of quantification of oligodendroglial cells (mature oligodendrocytes (Nogo-A+, APC+), proliferating OPC (Olig-2+/Ki-67+), reactive astrocytes (GFAP+), and activated microglia (Mac-3+ or RCA-1+) using cell-specific markers. Immunopositive cells with an identified nucleus (counterstaining with DAPI for immunofluorescence) were counted in the central part of the corpus callosum in an area of at least 0.185 mm^2^ using a magnification of ×400 (Olympus BX61) as previously described [56]. In the results, counted cells were expressed as the number of cells per mm^2^.

## 5. Conclusions

In conclusion, our results showed that CDP-choline promoted remyelination even at lower doses, which are similar to those usually applied in humans in clinical trials. Since CDP-choline has already been tested for other conditions in human clinical trials, the side effect profile is well known, and a fast translation of the hypothesis into a clinical study in patients with MS is feasible.

## Data Availability

Data is contained within the article.
